# Electrospun crosslinked poly-cyclodextrin nanofibers: Highly efficient molecular filtration thru host-guest inclusion complexation

**DOI:** 10.1038/s41598-017-07547-4

**Published:** 2017-08-07

**Authors:** Asli Celebioglu, Zehra Irem Yildiz, Tamer Uyar

**Affiliations:** Institute of Materials Science & Nanotechnology, UNAM-National Nanotechnology Research Center, Bilkent University, 06800 Ankara, Turkey

## Abstract

Water pollution is a serious concern for public health and environment in today’s world; hence, there exists a strong demand to develop cost-effective, sustainable and eco-friendly membranes. Here, we produce a highly efficient molecular filter membrane based on bio-renewable material; cyclic oligosaccaharides known as cyclodextrins (CD). Crosslinked insoluble poly-CD nanofibers are produced by using electrospinning technique in the absence of any additional polymeric carrier. Poly-CD nanofibrous membrane exhibit significant affinity to a common class of organic pollutant (i.e. methylene blue (MB)). Remarkably, the electrospun poly-CD nanofibrous web can outdistance the commonly used filter material (i.e. activated carbon) in terms of removal capacity. The flexible and free-standing poly-CD nanofibrous membrane depicted outstanding filtration performance. We estimate of above 90% removal efficiency for highly concentrated solutions of MB pollutant (40 mg/L) under extremely high flux (3840 Lm^−2^h^−1^). Essentially, these poly-CD nanofibrous webs demonstrate quite rapid uptake of MB from liquid environment. Overall, bio-based flexible electrospun poly-CD nanofibrous membrane represents a highly efficient molecular filter for wastewater treatment.

## Introduction

The toxic pollutants resulting from agricultural/industrial activities and fossil fuel combustion become the most important environmental concerns in today’s world. Organic dye molecules (textile dyes) create significant environmental hazard to clean water resources^1^. In this direction, several researches have been performed to develop convenient methods of purifying water with less input (cost, energy, chemical etc.) and load (ecologically disposing, recycling, reusing etc.) to the environment^2^. By the given objectives, ‘adsorption’ is the universally accepted method for robust and cost-effective decontamination of the effluents. Activated carbon (AC) is one of the most common adsorbent to remove organic contaminants from waste water; however, they are nearly indifferent to inorganic and hydrophilic pollutants^3^. The convenient use of AC is ensured only when integrated into a solid support. Additionally, energy intensive regeneration process of AC is another difficulty in practice^4^. The drawbacks in question lead demand for developing multi-purpose, easy-to-use, cost-effective and bio-based membranes. The fact that, electrospun nanofibers exhibit various unique properties such as high porosity, large surface area to volume ratio, high degree of interconnection and modifiable nature. These qualities make the nanofibers quite favorable candidate in filtration, separation and cleaning applications^5, 6^. Furthermore, these electrospun nanofibers can keep up with conventional filtration systems owing to their versatility and production variability.

Cyclodextrins (CD) are natural products from starch produced by means of enzymatic conversion. CD are cyclic oligosaccharides consisting of α(1,4)-linked glucopyranose units with a toroid-shaped molecular structure. CD are extensively used in separations, purification and filtration purposes due to their unique property to form non-covalent host-guest inclusion complexes (IC) including hazardous and polluting compounds^7, 8^. Hence, the filtration performance of electropsun nanofibers can be enhanced with the functionalization of CD inheriting its well-known host-guest encapsulation capability^9, 10^. Furthermore, handling and reusability problems of the powder and granular form of CD can be eliminated in filtration applications by the integration of CD functionality to the polymeric fibrous membranes. Several studies in the literature report on the encapsulation/attachment of CD within electrospun polymeric nanofibers for wastewater treatment and water filtration purposes^11–20^. However, if a polymeric matrix used as support material, the filtration performance of such CD functionalized nanofibers is limited significantly due to the presence of low fraction of CD moiety. Hence, nanofibers fully composed of CDs are more attractive. Contextually, we have firstly reported the electrospinning of CD nanofibers without any carrier polymeric matrix^21–24^. However, the water solubility of these CD nanofibers was an obstacle for water filtration which needs to be overcome. Therefore, the motivation of the present study is to develop more applicable and water-insoluble nanofibers based on CD. Notably, insoluble crosslinked granular CDs are already studied but with an unfortunate lower surface area to volume ratio and worse filtration performance compared to that of commercial AC^25^. Alsbaiee *et al*. enhanced the filtration efficiency of these granules by increasing its surface area to volume ratio (mesoporous). However, the granular form of these CD based adsorbent induced limitations such as additional preparation step during their application^4^. In the present study, to the best of our knowledge, we report very first and facile example of electrospinning of insoluble cross-linked poly-CD nanofibers as a flexible and self-standing nanofibrous filtering material for wastewater treatment. We further investigate the filtration performance of this poly-CD nanofibrous web under high flux conditions to demonstrate its practical application for water filtration. Contrary to CD functionalized polymeric nanofibers, poly-CD nanofibers are completely composed of CD molecules. Apart from its water-insoluble characteristic, the poly-CD nanofibrous membrane has shown outstanding removal capacity over the conventional filtering material (i.e. activated carbon) for organic dye pollutant, namely methylene blue (MB). Besides, poly-CD nanofibers are re-used subsequent to a mild washing procedure with almost the same performance, which is not the case for AC. Our results demonstrate that nanofibrous mats of electrospun poly-CD nanofibers having flexible and free-standing characteristics can be quite useful for rapid and efficient molecular filtration practices targeting a variety of organic pollutants in wastewater treatment.

## Results

### Fabrication of crosslinked poly-CD nanofibers

**Figure 1:**
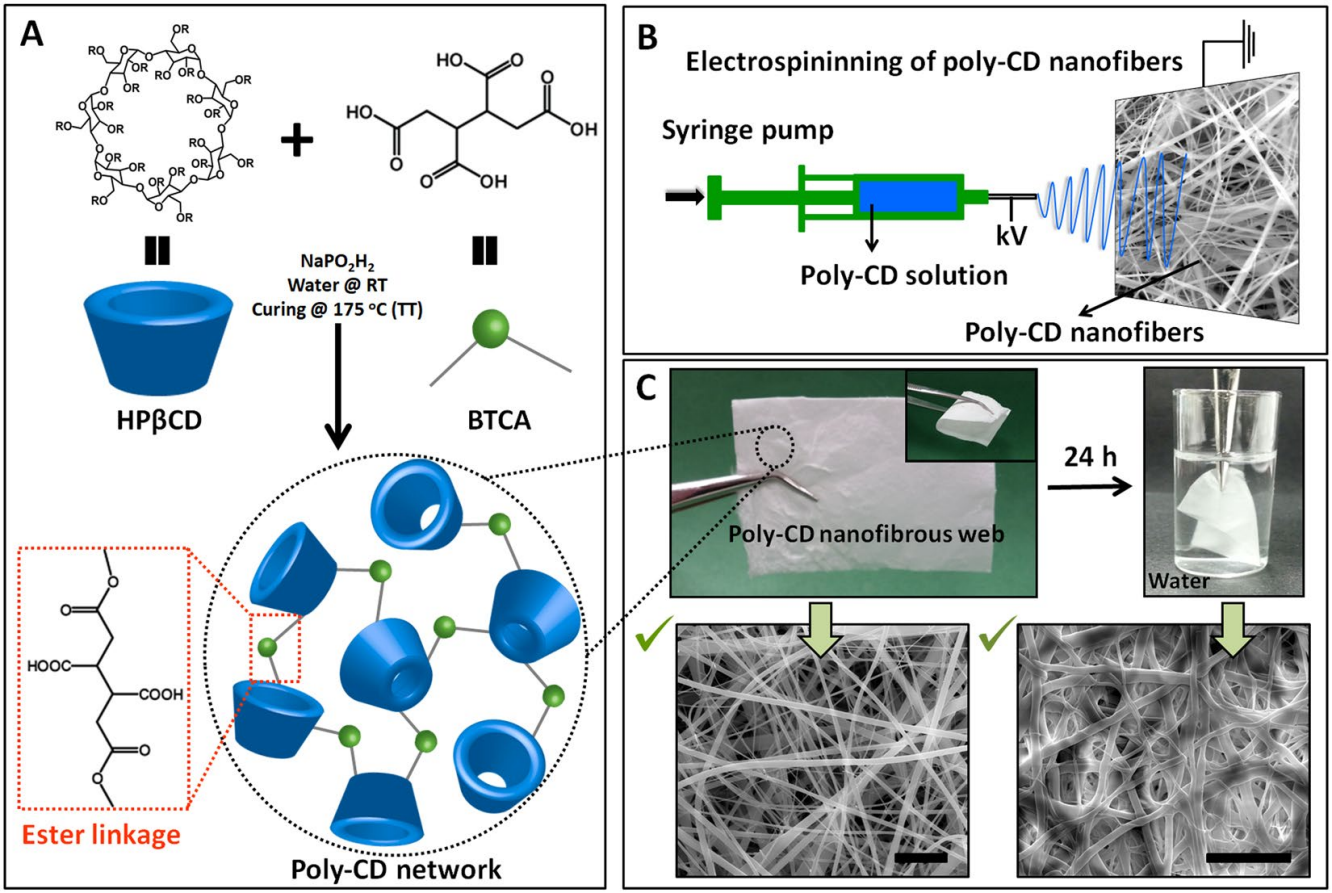
Fabrication of crosslinked poly-CD nanofibrous web. (**A**) Chemical structure and the schematic view of the HPβCD and BTCA. The schematic illustration of the cross-linked poly-CD network structure linked with ester linkage (randomly formed). **(B)** Schematic diagram of the electrospinning of the CD solutions containing BTCA and initiator as well. **(C)** Digital photograph of the self-standing feature and insoluble property of the poly-CD nanofibrous webs. The SEM images of poly-CD nanofibers before and after immersing in water for 24 h (scale bar-10 μm).

After rigorous optimization studies, the optimal electrospinning solution was prepared by mixing HPβCD, crosslinking agent and initiator at required levels. Initially, a known concentration of HPβCD was dissolved in water at room temperature. Then, crosslinking agent (BTCA) and initiator were added to the clear HPβCD solution (Fig. 1A). Once the solution is ready, electrospinning was carried out as the second step (Fig. 1B).The polymerization was completed with final thermal treatment yielding cross-linked poly-CD nanofibers. As it is depicted in Fig. 1A, crosslinked and insoluble poly-CD nanofibers were obtained from the esterification reaction between hydroxyl group of HPβCD and carboxyl moieties of crosslinking agent (BTCA). Although the polymerization of CD with BTCA has been reported previously^26^, the electrospinning of nanofibers from poly-CD without using a carrier polymeric matrix is unprecedented. It is anticipated that a network structure exists through the uniform fiber structure because of the uncontrolled reaction between crosslinking agent and CDs of hydroxyl groups (Fig. 1A). Based on this random network structure and CD being a small molecule to form the network in the fiber matrix, some brittle character may be expected for poly-CD nanofibers. However, self-standing poly-CD nanofibrous membranes were flexible having mechanical integrity (Fig. 1C). SEM analyses demonstrated the bead-free and homogenous morphology of poly-CD nanofibers with 160–1460 nm fiber diameter range (AFD: 480 ± 300 nm) (Fig. 1C). The insolubility of these nanofibers was checked by immersing them in water for 24 hours. Intact poly-CD nanofiber web (nanoweb) morphology is indicative of insoluble nature (Fig. 1C). The insoluble fraction of poly-CD nanofibers was determined to be almost 100% suggesting a successful crosslinking throughout the nanofibers. On the other hand, swelling analysis reveals a high water uptake capacity of 250 ± 17%, possibly due to the existence of polar carboxyl and hydroxyl groups in poly-CD structure which help to absorb water molecules. Nonetheless, the fibrous morphology of poly-CD membranes was preserved even after immersion in water for 24 hours. The wetting tendency of poly-CD nanofibrous mat is obvious from Fig. 1C. Additionally, the contact angle measurement for poly-CD nanofibrous mat confirmed the disappearing of water droplets in 0.02 s (Fig. S1). The high water uptake and fast wetting features of poly-CD nanofibers are desirable to improve the adsorbent performance and reduce pressure fluctuations during the filtration processes^27^. In this study, we aim to obtain water-insoluble poly-CD nanofibers for water filtration, nevertheless, the insolubility and durability of the poly-CD nanofibrous webs against organic solvent were also confirmed after keeping them in various solvents (DMF, DMSO, ethanol, methanol, acetonitrile, chloroform, acetone etc.) for 24 hours (Fig. S2). Hence, the stability of poly-CD nanofibers in common organic solvents may potentially provide greater opportunities as a filtering material in organic liquid mediums as well.

### Characterization of poly-CD nanofibers

**Figure 2:**
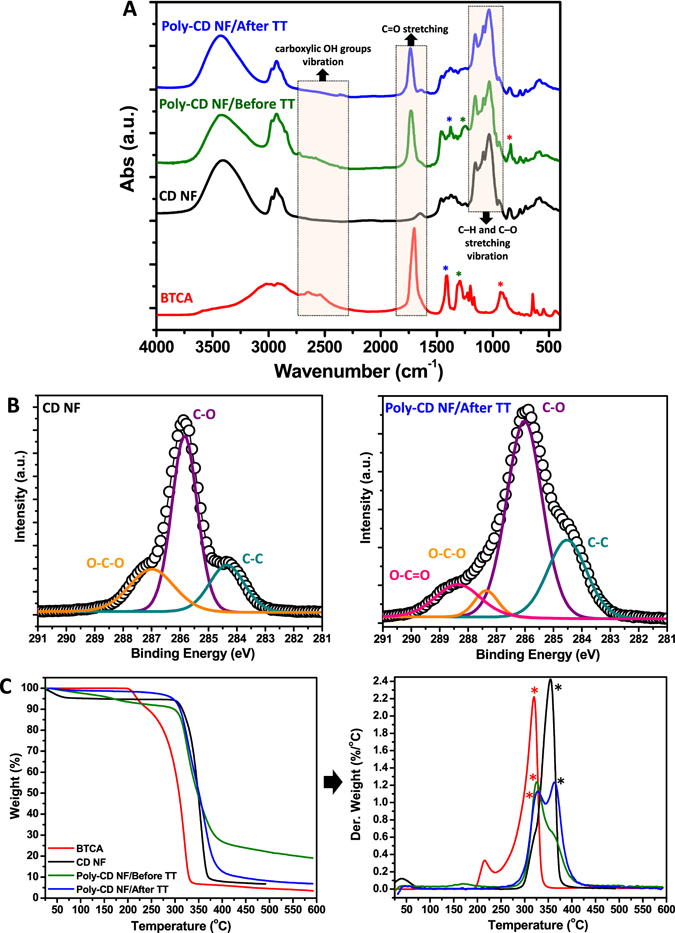
Characterization of cross-linked poly-CD nanofibers. (**A**) FTIR spectra of BTCA, pure CD nanofibers (NF), poly-CD NF before and after thermal treatment (TT). The prominent absorption bands (C-H and C-O stretching vibration) of CD are also present for poly-CD NF. The broad absorption band at 2600–2700 cm^−1^, corresponds to the vibration of H-bonded carboxylic OH groups of BTCA, disappear after TT along with the formation of ester linkage. The strong peak of C=O stretching at 1703 cm^−1^ shifts to higher wavenumbers for poly-CD NF suggesting the esterification reaction between CD and BTCA molecules. The other characteristic peaks of BTCA also (assigned as “***”) disappear after TT due to crosslinking. (**B**) The high resolution C 1 s XPS spectra of pure CD NF and poly-CD NF after TT. The spectra of the pure CD NF is deconvoluted into three peaks (assigned to C–(C, H, C-O and O-C-O). A new peak is observed in the case of poly-CD NF/after TT which is belong to O=C-O of carboxyl/ester groups. (**C**) TGA thermograms of BTCA, pure CD NF, poly-CD NF before and after TT. There is an additional degradation step of BTCA at *325 °C before TT, and it becomes indistinct. The degradation step of CD moiety (*365 °C) appears after TT as the crosslinker units form the final structure of poly-CD network.

Analyses of FTIR spectra revealed the formation of poly-CD as a result of cross-linking process. Here, the structure of poly-CD nanofibers was compared with the water-soluble non-polymeric pure CD nanofibers. The FTIR spectra of pure CD and poly-CD nanofibers before and after thermal treatment (TT) are shown in Fig. 2A. HPβCD depicted prominent absorption bands at about 1155, 1082 and 1035 cm^−1^ owing to C-H and C-O stretching vibration^28^. Similar absorption bands were also seen in poly-CD nanofibers. The degree of cross-linking was determined from the absorbance ratio (*A*_1082_/*A*_1033_) in this region of the IR spectrum. After thermal treatment, an increase of about 66% was observed in the *A*_1082_/*A*_1033_ ratio for poly-CD nanofibers when compared to pure CD nanofibers. This increase indicates that around 66% (approximately two thirds of OH groups) of the primary OH of CD transformed into secondary OH forming the cross-linking junction^29^. In addition to this, the broad absorption band between 2600–2700 cm^−1^ (H-bonded carboxylic OH groups of BTCA) disappear after thermal treatment which suggests the esterification reaction between CD and BTCA molecules. The strong peak of BTCA at 1703 cm^−1^ is attributed to C = O stretching of the carboxyl groups^30^. The band at 1703 cm^−1^ from pure BTCA shifts to 1729 cm^−1^ before TT which shifts to 1736 cm^−1^ after TT apart from esterification (Fig. 2A). XPS measurements further support the results obtained by FTIR spectroscopy. It was determined that C 1 s and O 1 s are two intensive elements as the main compositions of the CD based nanofibers. The high resolution C 1 s spectrum from pure CD nanofibers can be deconvoluted into three peaks assigned to C-(C, H) at 284.4 eV, C-O at 285.8 eV, O-C-O at 287.0 eV (Fig. 2B)^20^. In the case of poly-CD nanofibers after TT, additional peak at 288.4 eV is observed which was attributed to O=C-O of carboxyl/ester groups (Fig. 2B)^31^. The corresponding positions of peak binding energies and their values (% area ratio) are also listed in Table S1. After thermal treatment, the C 1 s spectrum (Fig. 2B) clearly shows a decrease of O-C-O along with an increase of intensity from that of from O=C-O functional group (Table S1). Furthermore thermal and structural properties were studied with TGA and XRD, respectively. In TGA thermogram of poly-CD nanofibers, we noted an additional degradation step at 325 °C from BTCA before TT which becomes indistinct after TT. Since the crosslinker become a part of poly-CD network after the reaction, the degradation step of CD moeity (365 °C) also appears signally after TT (Fig. 2C). Further studies by XRD revealed amorphous nature of non-polymeric pure CD nanofibers and poly-CD nanofibers with a similar broad diffraction pattern (Fig. S3). The absence of distinctive diffraction peaks is attributed to the lack of definite orientation between CD molecules in both pure CD fibers and poly-CD structures. In addition, the sharp diffraction peaks from BTCA (Fig. S3) are not observed in poly-CD nanofibers which indicates the reaction homogenity of the BTCA crosslinker with the CD molecules in the poly-CD matrix. It may be anticipated that, poly-CD nanofibrous webs are fragile due to its cross-linked structure, however, as it is also observed in Fig. 1C, these nanowebs have shown mechanical integrity and flexibility. Further, the mechanical property of the poly-CD nanowebs was investigated by using tensile test. Table S2 summarizes the mechanical properties of these nanofibrous membranes which were obtained from the stress-strain curve. It was observed that the ultimate tensile strength and the young modulus of poly-CD nanoweb are quite higher when compared to non-polymeric pure CD nanofibrous web (Table S2). The higher mechanical property for poly-CD nanoweb is attributed to the formation of crosslinked network structure. Eventually, poly-CD nanowebs are self-standing which can be easily folded and handled enabling their use in a filter module without any support. By N_2_ physisorption test, the BET surface area and porosity of poly-CD nanowebs were also investigated. The poly-CD nanoweb has 6.45 m^2^/g surface area; 0.16 cm^3^/g total pore volume and 85.60 nm average pore width (Table S3). These values are close to those values of electropsun polymeric nanofibrous webs reported in the literature^32, 33^.

### Adsorption kinetics

**Figure 3:**
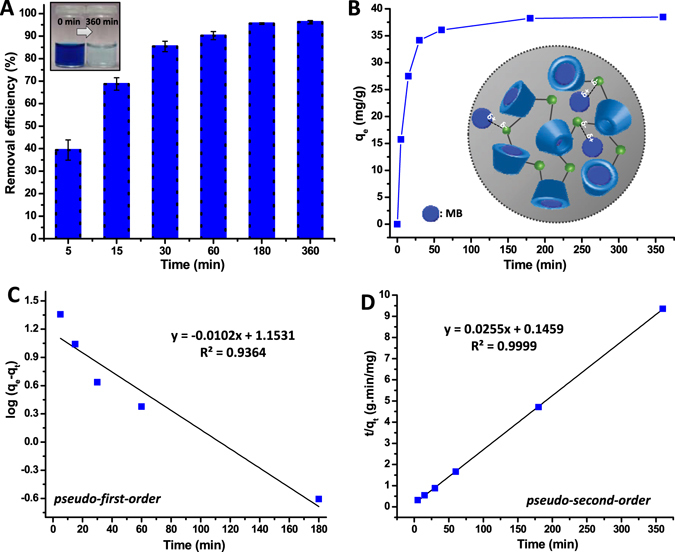
Adsorption kinetics and mechanism of MB by poly-CD nanofibrous membrane. (**A**) Time dependent removal efficiency of MB (initial concentrations of 40 mg/L) by poly-CD nanofibrous membrane (n = 3). (**B**) Adsorption kinetics of MB by poly-CD nanofibrous membrane plotted in terms of q_e_ values. Schematic representation of the inclusion complexation of MB molecules with CD cavities within the poly-CD matrix. Additionally, the illustration of possible electrostatic interaction of MB with poly-CD matrix. **(C)** Pseudo-first-order kinetic plots and **(D)** pseudo-second-order kinetic plots of MB. The adsorption of MB is better modelled with second-order kinetics than first-order kinetics.

Methylene blue (MB) is a widely used dye in industries which has a potential risk such as mutagenic/carcinogenic effects and bioaccumulation even at very low concentrations^1, 12, 13^. During the adsorption of MB by poly-CD nanoweb, inclusion complexes and electrostatic interactions could occur in the course of time. So, progressing time intervals would be crucial to follow the adsorption kinetics of MB by poly-CD nanofibrous membrane (Fig. 3). Therefore, the adsorption kinetics of MB on poly-CD nanofibers was investigated with an initial concentration of 40 mg/L. Figure 3 shows the removal efficiency (%) over time. Relatively faster adsorption process was observed within the first 5 min due to the existence of several active adsorption sites. Notably in the first 15 min ~70% of MB was removed from its aqueous solution (Fig. 3A). The adsorptions reached saturation by 360 min which was chosen as the equilibration time for the subsequent tests. At this stage the equilibrium uptake reached 96.2 ± 0.8% (Fig. 3A). Kinetic studies provide critical insight into the rate of adsorption processes therein. The pseudo-first-order and pseudo-second-order models are applied to investigate the adsorption kinetics behavior of poly-CD nanofibers. Figure 3C and D show such plots and Table S4 summarizes the kinetic adsorption parameters calculated from these two models. The adsorption process of MB is better represented by pseudo-second-order model with regards to correlation coefficient (R^2^). This indicates that the chemical interactions are effectual during the adsorption process, while for second-order kinetic model the equilibrium adsorption capacity (q_e_) is 39.17 mg/g which is correlated with experimental adsorption capacity (q_exp_) (Table S4). Additionally, we have observed that, poly-CD nanofibers have shown much higher adsorption capacity of MB from aqueous environment when compared to that of a latest report on CD functionalized nanofibers^12, 34^ (detailed comparison with the literature is given in note S1). The electrostatic interaction between poly-CD nanofibers and MB is also possible although inclusion complexation of MB with the CD′s cavity plays a major role for the removal of MB (Fig. 3B)^12^.

### Adsorption equilibrium isotherm

**Figure 4:**
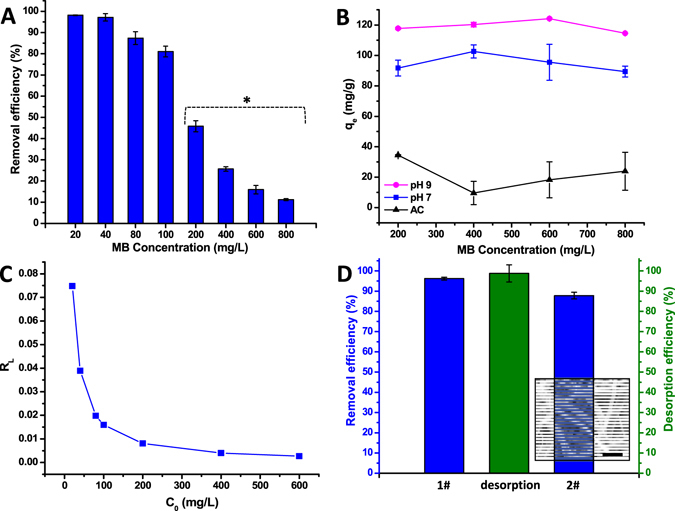
Removal efficiency, adsorption capacity of poly-CD nanofibrous membrane and comparison with activated carbon (AC) and reusability of poly-CD membrane. (**A**) MB removal efficiency (%) of poly-CD membrane against concentration. The adsorbed amount of MB decreases gradually by the increasing dye concentration (n = 3). (**B**) Comparative adsorption capacity (q_e_ (mg/g)) graphs of poly-CD membrane (for pH = 7 and pH = 9 MB solutions) and AC are plotted for four different MB concentrations (*200–800 mg/L). Poly-CD membrane (pH = 7) indicate significantly higher capacity compared to AC. The adsorbed MB amount can be drawn up easily by adjusting the pH = 9 (n = 3). (**C**) R_L_ graphs for the adsorption of MB indicating the high tendency between MB and adsorbent, and favorable uptake process. (**D**) The reusability of poly-CD membrane. The desorption of MB from poly-CD membrane is achieved by washing with methanol solution containing 5% (v/v) HCl. The practical recovery is provided and poly-CD membrane offers almost the same adsorption potential for the second cycle. The inset SEM image of poly-CD membrane after reusability tests (scale bar-10μm) confirms that the fiber morphology of poly-CD membrane is still preserved after the adsorption tests (n = 3).

Here, the adsorption results were fitted with two well-known Langmuir and Freundlich models to evaluate the equilibrium isotherms^35^. The resulting equilibrium parameters are summarized in Table S5 and the plots were displayed in Fig. S4. Langmuir model proposes a relatively homogeneous and monolayer adsorption, in contrast Freundlich model predicts heterogeneous surfaces and multilayer sorption^13, 35^. Comparison of correlation coefficients (R^2^ = 0.9983) indicated that adsorption results are better fitted with Langmuir than that of Freundlich model. This suggests the homogeneous monolayer adsorption of dye molecules with 97.4 mg/g (q_max_) equilibrium capacity. On the other hand, the increased pH condition in fact enhanced the adsorption capacity (124.1 mg/g) of poly-CD nanofibers. This is due to the decreased protonation of poly-CD, increased electrostatic interaction and host-guest inclusion complexation^12, 13^. Therefore, more MB could be removed by poly-CD nanofibers where heterogeneous and multilayer adsorption kinetics are expected. Here, we have tested commercial AC for q_e_ value as a control sample. Figure 4A depicts removal efficiency of poly-CD nanofibers for various MB concentrations. Figure 4B depicts q_e_ values of poly-CD nanofibers (for pH = 7 and pH = 9) and AC with reference to MB concentrations. The progressively darker color of poly-CD nanofiber is associated with the incremental concentration of MB solutions (Fig. S5). Although AC (600–800 m^2^/g) depicted extremely higher surface area than that of poly-CD nanoweb (6.45 m^2^/g), MB removal capacity of the poly-CD nanoweb (q_max_ = 96.79 mg/g) is remarkably higher than that of AC (q_max_ = 14.73 mg/g), see Fig. 4B. This finding exploits the formation of inclusion complex with CD cavity and therefore enhances dye removal capacity. Moreover, dimensionless separation factor R_L_ was calculated by using Langmuir model which turns out to be 0 < R_L_ < 1. This reveals the high affinity between pollutant and adsorbent apart from favorable uptake of MB (Fig. 4C)^13^. The reusability is of vital concern for adsorbent materials to reduce the cost and environmental loading. Unlike AC which requires high energy consumption for regeneration, the adsorbed pollutants can be easily removed from poly-CD nanofibers via a simple washing process. Here, the entrapped MB molecules were desorbed into a methanol solution containing 5% (v/v) HCl. The removal efficiency is ∼ 90% for the reused poly-CD nanofibrous membrane (Fig. 4D). The structural stability of poly-CD nanofibers was inspected after reusability tests. The SEM imaging suggested that the poly-CD membrane maintained its fibrous structure even after the adsorption experiments which include mechanical forces (Fig. 4D, inset).

### Filtration performance of poly-CD nanofibrous membrane

**Figure 5:**
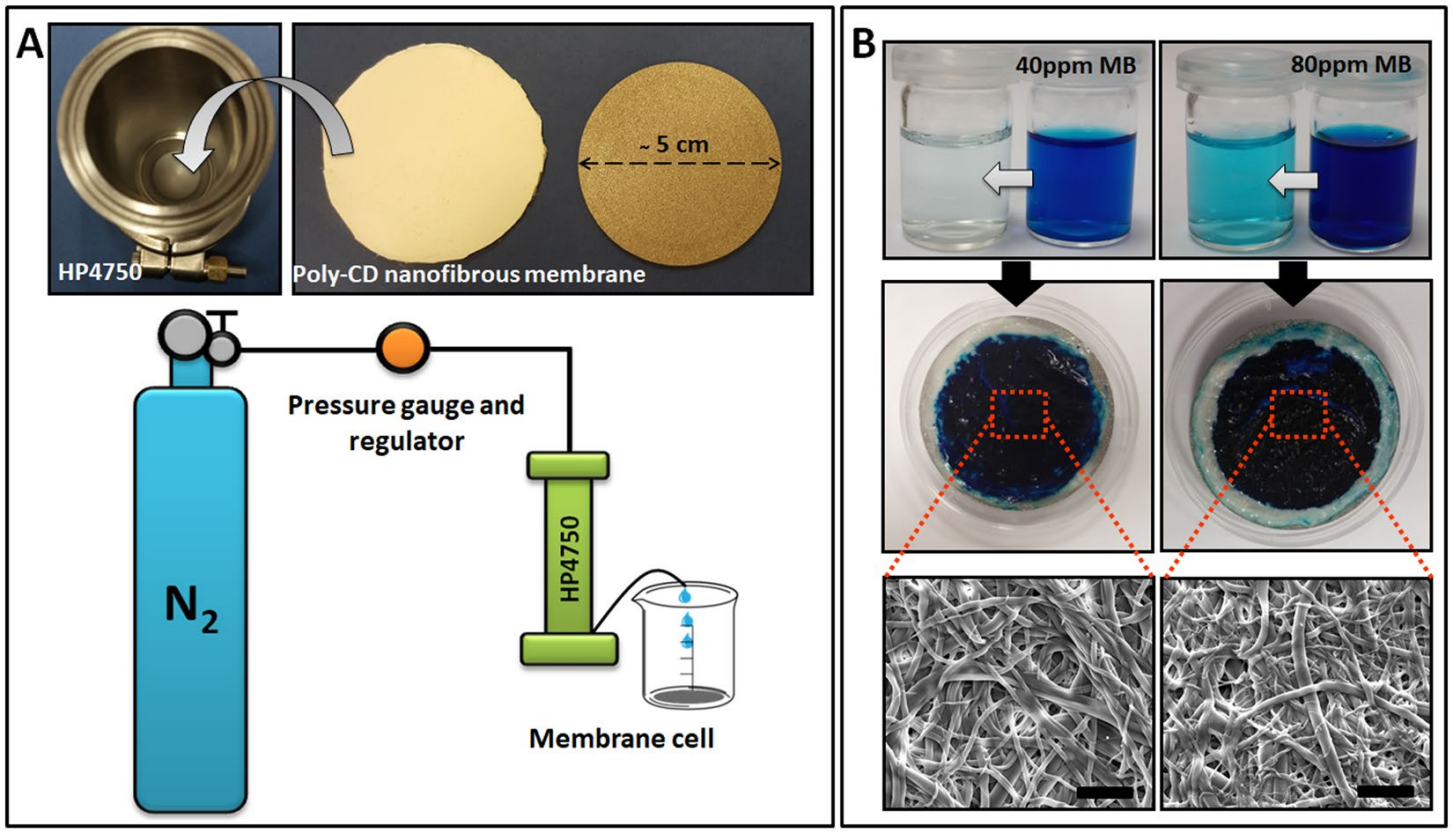
Filtration performance of poly-CD nanofibrous membrane. (**A**) The photographs of membrane cell part of HP4750 dead-end system and the cropped poly-CD nanofibrous membrane with a definite active filtration area (14.6 cm^2^). The schematic view of HP4750 filtration system. For each test, 50 mL solution is passed through the poly-CD nanofibrous membranes with a definite N_2_ pressure. Then, the permeated solution is collected in a clear beaker. (**B**) The visual illustration of the MB solutions prepared at two different MB concentrations (40 and 80 mg/L) before and after filtration test. The photographs and SEM images (scale bar-10 μm) of the poly-CD nanowebs exposed to these two concentrated MB solutions during the experiments. As clearly seen, both the macroscopic visual appearance and the fibrous morphology of poly-CD nanofibers were protected under such applied pressure.

The poly-CD nanofibrous web is uniquely suited to be used as filter membrane due to its free-standing and flexible properties. We further investigated the dynamic filtration performance of poly-CD membrane by dead-end filtration system. For these experiments, poly-CD membranes were used as a thin layer in a membrane cell of HP4750 (Sterlitech) system. 50 mL of MB aqueous solution (40–80 mg/L) was passed rapidly through the poly-CD nanofibrous membranes (active filtration area; 14.6 cm^2^) by applying a known N_2_ pressure (10 kPa) (Fig. 5A). Operating pressure is a key factor for the performance of membrane. Therefore, for trial purposes two pressure values, viz 100kPa and 10kPa were initially tested on 40 mg/L MB solutions to evaluate the filtration differences due to pressure variations (Fig. S6). Since the penetration time of the solution through the poly-CD membrane decreases for higher pressures, the solution flux significantly increases (Fig. S6). Correspondingly, this lessened the removal efficiency of MB. Hence, we have employed 10 kPa pressure for the rest of the experiments (Table S6). In this case, lower flux could not be considered as a shortcoming as increased contact time supports the adsorption process. Under this condition, poly-CD membrane removed 98.6 ± 0.4% and 95.6 ± 0.9% of MB from 40 and 80 mg/L solutions, respectively (Fig. 5B) (movie S1). The ultimate blue color of poly-CD membranes demonstrated a good adsorption or separation process. The obtained results showed that the poly-CD nanofibrous membranes can filter organic dye pollutant with higher efficiency than equilibrium uptake values. This dynamic filtration was tested for a very short time (30–40 s, 50 mL stock solution). This outstanding performance of poly-CD nanofibrous membrane suggests that CD cavities and moieties are mostly accessible allowing a fast retention of MB during actual filtration practices. Permeability and flux are two critical parameters to evaluate the filtration performance of the membranes. In this study, the dead-end filtration system enabled us to determine these two criteria and manifest real-like results differently from previous reports in the literature^12, 13^. Table S6 summarizes the average permeability and flux values for poly-CD nanowebs. Associated with the batch variations, the permeability and flux of poly-CD membrane were calculated in the range of 237–291 Lm^−2^h^−1^kPa^−1^ and 3561–3842 Lm^−2^h^−1^, respectively. However, flux fluctuations were not observed during the filtration process. Here, poly-CD membranes were tested with a generally used thickness of ∼ 1 mm, in which they showed extremely higher permeation and flux compared to the reported membranes^35, 36^.This is probably due to their highly porous structure and hydrophilicity^35, 36^ which favor the water flow and enhancing the permeability of membrane and filtration efficiency. Additionally, the pores of nanofibrous web most probably create pseudo-channel structure within the membrane which provides an efficient pressure distribution preventing pressure fluctuations that can reduce the membrane performance^27, 35^. In addition to high permeability, the acceptable salt rejection (∼ 93%) and the low B/A ratio (salt permeability/water permeability) were achieved by poly-CD nanofibrous membrane (Fig. S7)^37, 38^. As in the batch adsorption studies, the nanofibrous morphology of poly-CD membrane was preserved after filtration and macroscopic visual appearance of nanoweb was not destroyed even under applied pressure (Table S6). Overall, these promising results strongly support our idea to exploit these electrospun poly-CD nanofibrous webs for water filtration purposes.

## Discussion

In this study, we produced insoluble poly-CD nanofibrous web from HPβCD, crosslinking agent and initiator combination by using electrospinning technique. The morphological SEM characterization displayed the insolubility and stable fiber morphology of the poly-CD webs in water and several organic solvents. The swelling test and contact angle measurement demonstrated the highly hydrophilic nature of poly-CD nanofibers which is in great demand for filtration applications. After successful optimization steps, we initially proved the formation and existence of cross-linked poly-CD nanofiber structures via compositional/thermal analysis carried out by FTIR, XPS and TGA. It was also found that, approximately two thirds of OH groups of CD contributed to cross-linking junctions in poly-CD nanofiber matrix. Further characterization indicated that the poly-CD nanofibers have similar amorphous structure as that of non-polymeric pure CD nanofibers, yet, the poly-CD webs depicted better mechanical properties than the non-polymeric pure CD nanofibers due to their crosslinked network structure. Due to the influential concept of poly-CD membrane, the applicability of the CD molecules is significantly enhanced providing flexible and free-standing characteristics. These features are rather similar to that of commercial membranes. Furthermore, higher surface area to volume ratio and porosity of electrospun nanofibrous webs are notable advantages. The ultimate structure of the poly-CD nanofibrous webs is found to offer efficient removal capability for organic dye pollutants. Poly-CD nanofibrous membranes exhibited excellent affinity to MB contaminants and showed the removal capability from aqueous environment with high adsorption capacity. More importantly the tested MB concentrations are extremely higher than the acceptable limits of the concerned pollutant quantified in water (according to the U.S. Environmental Protection Agency (EPA)^39^. AC was selected as a control commercial sample for the comparative adsorption studies. Remarkably, poly-CD nanofibrous webs have shown greater removal performance than that of AC. Contrary to energy intense regeneration procedure of AC, the poly-CD nanofibers indicated a successful reusability after a simple washing process. Moreover, poly-CD nanofibrous membrane was tested in dead-end filtration system for the dynamic filtration experiments. The filtration processes were carried out under definite pressure and solution flow at high flux values. The electrospun poly-CD nanofibrous webs have shown rapid and effective uptake of organic dye pollutant. This demonstrates the feasibility of such novel membranes for practical filtration applications. To conclude, electropsun poly-CD nanofibers, which are simply originated from bio-renewable material such as starch, can be utilized as an efficient filter membrane for the removal of organic dye pollutants from wastewater.

## Methods

### Materials

Hydroxypropyl-β-cyclodextrin (HPβCD), molar substitution: ∼ 0.6), was purchased from Wacker Chemie AG, Germany. N,N-dimethylformamide (DMF) (Rie-del, Pestenal), acetonitrile (ACN) (Chromasolv, HPLC ≥99.9%), isopropyl alcohol (IPA), ethanol (Sigma-Aldrich ≥99.8% (GC)), methanol (Sigma-Aldrich ≥99.7% (GC)), chloroform (Sigma-Aldrich 99–99.4%) (GC)), acetone (Sigma-Aldrich, ≥99% (GC)), tetrahydrofuran (THF), dichloromethane (DMC), dimethyl sulfoxide (DMSO) (Sigma-Aldrich, 99.9%), potassium bromide (KBr, Sigma-Aldrich, 99%, FTIR grade), 1,2,3,4-butanetetracarboxylic acid (BTCA) (Sigma Aldrich, 99%), sodium hypophosphite hydrate (SHP) (Sigma Aldrich), methylene blue (MB) (Sigma-Aldrich, ≥82%) and activated carbon (AC) (Sigma-Aldrich, untreated, granular, 8–20 mesh) were obtained commercially. The water used was from a Millipore Milli-Q Ultrapure Water System. All the materials were used without any purification.

### Electrospinning of poly-CD nanofibers

The optimum solution system, electrospinning parameters and thermal treatment conditions for the production of poly-CD nanofibers were determined by testing various different combinations. As an optimized scheme, a known concentration of HPβCD (140% w/v) was dissolved in water at room temperature. As the HPβCD dissolved, BTCA was added to the clear HPβCD solution at 20% (w/w, according to CD). Then 2% (w/w, according to HPβCD) of initiator (SHP) was added to CD-BTCA solution. The temperature of the mixture was kept at 50 °C under continuous stirring until the viscosity of solutions reached to critical points. Once the system was cooled down to room temperature the electrospinning was carried out. Solutions were taken in a syringe (1 mL) with a metallic needle with an inner diameter of 0.45 mm attached. Thereafter the syringe was positioned horizontally on a syringe pump (Model: KD Scientific, KDS-101). The electrode of the high voltage power supply (Matsusada Precision, AU Series) was clamped to the metal needle tip and ground to an aluminum collector which was wrapped with an aluminum foil. The electrospinning of the solution was performed with the following parameters. Applied voltage = 10 kV, tip-to-collector distance = 10 cm and the solution flow rate = 1.0 mL/h. The electrospinning apparatus was enclosed in a Plexiglas box and the electrospinning was carried out at 22–26 °C at 25–30% relative humidity. Finally, the crosslinked and insoluble poly-CD nanofibers were obtained with thermal treatment (TT) where the curing reaction took place in oven at 175 °C for 1 hour. The un-reacted parts, if any, were removed by washing the thermally treated poly-CD nanofibrous webs with water and ethanol/acetonitrile. The solvent durability of electrospun poly-CD webs was investigated by soaking the samples in water, acetonitrile, isopropyl alcohol, ethanol, methanol, chloroform, acetone, tetrahydrofuran, dichloromethane, dimethylformamide and dimethyl sulfoxide for 24 hours. For a comparative characterization of the poly-CD nanofibers, non-polymeric pure CD nanofibers were also obtained. The water-soluble non-polymeric pure CD nanofibers was prepared as reported in our previous work^22^. The highly concentrated solution of HPβCD (160% w/v) was dissolved in water at room temperature without an additional crosslinking agent, then pure CD nanofibers was produced via electrospinning by using the above mentioned electrospinning process parameters.

### Characterization

The morphological characterization of nanofibers was carried out by scanning electron microscope (SEM) (Quanta 200 FEG, FEI). Samples were sputter coated with 5 nm Au/Pd (PECS-682) alloy and the average fiber diameter (AFD) and fiber diameter range were calculated from the SEM images by analyzing around 100 fibers. Swelling behavior of poly-CD nanofibrous web was evaluated by calculating the absorbed water amount by the poly-CD web sample at room temperature (25 °C). First, the dried mass (W_0_) of nanofibrous membrane was measured. Then the samples were kept in distillated water for 24 h and weights of the swollen samples (W) were measured after removing the excess of water from the surface of the membranes with a filter paper. The swelling degree (Q) was calculated using the following formula:

1 $${\rm{Q}}=({\rm{W}}-{{\rm{W}}}_{0})/{{\rm{W}}}_{0}\times 100$$

For the solubility experiment, squares of 1 cm^2^ of the poly-CD nanofibrous webs were immersed in deionized water. After 24 h, the poly-CD webs were removed from the deionized water and placed in a vacuum oven to dry until they reach a stable weight. The insoluble fraction (%) is determined by following formula:

2 $${\rm{Insoluble}}\,{\rm{fraction}}( \% )={{\rm{W}}}_{{\rm{i}}}/{{\rm{W}}}_{0}\times 100$$

where W_0_ and W_i_ are the weight of the initial and after drying, respectively. Contact-angle meter (OCA 30, Dataphysics) was used for the contact angle measurements. Fourier transform infrared spectrometer (FTIR) (Bruker-VERTEX 70, Germany) was used to obtain the infrared spectra of samples. For this, the samples were mixed with KBr and pressed as pellets. 64 scans were recorded between 4000 and 400 cm^−1^ at a resolution of 4 cm^−1^. XPS measurements (Thermo Scientific, K-Alpha, monochromatic Al Kα X-ray source, 400 μm spot size, hν = 1486.6 eV) were performed in the presence of a flood gun charge neutralizer. For high resolution spectra, Avantage software was used for the peak deconvolution. Thermal characterization was performed by using thermogravimetric analyzer (TGA) (Q500, TA Instruments). The TGA of the samples were carried out from 25 to 500 °C at 20 °C /min heating rate with N_2_ as a purge gas. XRD data were obtained (2θ = 10–30°) by employing a PANalytical X’Pert Multi Purpose X-ray diffractometer with Cu Kα radiation (λ = 1.5418 Å). Tensile tests for poly-CD nanofibrous webs were carried out by using dynamic mechanical analyzer (DMA, Q800 TA Instruments) equipped with a tensile fixture. The stress/strain curves of rectangular shaped poly-CD web samples were obtained at 0.25 N/min force ramp and the average values were calculated by performing three measurements. For each sample, the gap between jaws was kept at 7 mm and the responses were recorded at room temperature. BET surface area analyzer (A Micromeritics Tristar 3000) was used to determine the surface area, average pore diameter and cumulative pore volume of nanofibrous webs. Nitrogen adsorption isotherm data were collected at 77 K. Prior to the analysis; the sample was degassed for 24 h at 373 K. BJH (adsorption) (Barrett, Joiner and Halenda) method was used to characterize the porosity of poly-CD nanofibrous webs. The UV-vis spectrophotometer (Varian Cary 100, USA) was used in the wavelength range of 400–800 nm to evaluate the absorbance of methylene blue (MB) solutions during the dye removal test. The concentration of MB solutions was determined according to calibration curve formed in the range of 20–800 mg/L MB concentration with R^2^ ≥0.99 acceptability.

### Batch adsorption studies

Batch adsorption tests were performed on a shaker (IKA KS 130 basic, Germany) at 160 rpm. Activated carbon (AC) (BET = 600–800 m^2^/g, 8–20 mesh, untreated) was used as a control sample. Initially, 5 mg of poly-CD webs were immersed in 40 mg/L aqueous solution (5 mL) of MB for adsorption kinetics. Then, the adsorption capacity was evaluated for initial concentrations ranging from 20–800 mg/L by using the same parameters (5 mL MB solution/5 mg poly-CD). Additionally, the pH value of MB solution was adjusted to basic (~pH 9) by adding 0.1 mol/L NaOH solution dropwise. To compare the effect of pH on AC, the adsorption tests were performed in the range of 200–800 mg/L MB concentration. MB-adsorbed poly-CD nanofibrous membranes were washed with methanol solution containing 5% (v/v) HCl for the desorption experiment. These poly-CD nanofibrous membranes were reused in adsorption experiments (adsorbent, 5 mg; MB, 5 mL, 40 mg/L). The concentrations of MB solutions were determined by UV–vis spectroscopy based on the standard curve. The efficiency of pollutant removal (%) by the adsorbent was calculated with the following formula:

3 $${\rm{Removal}}\,{\rm{efficiency}}( \% )=({{\rm{C}}}_{0}-{{\rm{C}}}_{{\rm{t}}})/{{\rm{C}}}_{0}\times 100$$

where C_0_ and C_t_ are the initial and residual concentration of pollutant in the stock solution and filtrate, respectively. The adsorption capacity (q_e_) of adsorbents was determined with the following equation

4 $${{\rm{q}}}_{{\rm{e}}}({\rm{mg}}/{\rm{g}})=({{\rm{C}}}_{0}-{{\rm{C}}}_{{\rm{e}}})\times ({\rm{V}}/{\rm{W}})$$

where C_0_ and C_e_ are the initial and the equilibrium concentrations of pollutant in the test solution (mg/L), V is the volume of the testing solution (L), and W is the weight of the adsorbent (g). Pseudo-first and second-order models are used to investigate the adsorption kinetics with the equations 5 and 6, respectively.

5 $$\mathrm{log}({{\rm{q}}}_{{\rm{e}}}-{{\rm{q}}}_{{\rm{t}}})={{\rm{logq}}}_{{\rm{e}}}-{{\rm{k}}}_{1}{\rm{t}}/2.303$$

6 $${\rm{t}}/{{\rm{q}}}_{{\rm{t}}}=1/{{\rm{k}}}_{2}{{{\rm{q}}}_{{\rm{e}}}}^{2}+{\rm{t}}/{{\rm{q}}}_{{\rm{e}}}$$

where q_t_ and q_e_ (mg/g) are the adsorption capacity at time t and equilibrium, respectively, and k_1_ (min^−1^) and k_2_ (g/mgmin) are the first and second-order rate constants, respectively. The equilibrium isotherms are studied with Langmuir and Freundlich models.

Langmuir isotherm:

$${{\rm{C}}}_{{\rm{e}}}/{{\rm{q}}}_{{\rm{e}}}=1/{{\rm{bq}}}_{{\rm{m}}}+{{\rm{C}}}_{{\rm{e}}}/{{\rm{q}}}_{{\rm{m}}}$$

Freundlich isotherm:

$${{\rm{lnq}}}_{{\rm{e}}}={{\rm{lnK}}}_{{\rm{F}}}+1/{\rm{n}}({{\rm{lnC}}}_{{\rm{e}}})$$

where q_e_ is the equilibrium adsorption capacity of the pollutant adsorbed onto the adsorbent (mg/g), C_e_ is the equilibrium concentration of the adsorbate (mg/L), and q_m_ and b are Langmuir constants associated with maximum adsorption capacity and binding energy, respectively. K_F_ and n are empirical constants where the former is known as Freundlich constant (L/mg) and the latter to be heterogeneity factor.

In addition, using the Langmuir parameters the dimensionless separation factor R_L_ was calculated with the following formula

7 $${{\rm{R}}}_{{\rm{L}}}=1/(1+{\rm{b}}\times {{\rm{C}}}_{0})$$

where b is the Langmuir constant (L/mg), and C_0_ is the highest initial pollutant concentration (mg/L). This factor represents the affinity between adsorbate and adsorbent where R_L_ >1 − unfavorable, R_L_ = 1 − linear, 0 < R_L_ < 1 −  favorable or R_L_ = 0 − irreversible. All experiments were repeated three times.

### Filtration test of poly-CD nanofibrous membranes

Dead-end filtration system (Sterlitech HP4750) was used to evaluate the filtration performance of poly-CD nanofibrous membrane. It consists of a 300 mL stainless steel cell which can be pressurized with N_2_ gas. The active membrane surface area is about 14.6 cm^2^. The purge pressure of the cell is adjusted by using a pressure gauge and regulator. For filtration tests, poly-CD membranes were cut into 5 cm diameter discs and placed in dead-end cell. Before the filtration experiments, prepared membranes were conditioned and pressurized with distilled water at 10kPa pressure to attain equilibrium and to reach reliable results. Afterwards, the cell was filled with 50 mL test pollutant (MB) solution and operation pressure was maintained at ~10kPa. The concentrations of MB solutions were arranged in the range of 40–80 mg/L. The experiments were performed at 25 °C and the filtered solutions were collected in clean beakers for further analysis. Each experiment was performed in triplicate and average value was reported. The flux (F) and water permeability (P_w_) of poly-CD membranes were calculated by using equations 8 and 9, respectively.

8 $${\rm{F}}({{\rm{Lm}}}^{-2}{{\rm{h}}}^{-1})={{\rm{V}}}_{0}({\rm{L}})/{{\rm{A}}}_{0}({{\rm{m}}}^{2})\times {{\rm{T}}}_{0}({\rm{h}})$$

9 $${{\rm{P}}}_{{\rm{w}}}({{\rm{Lm}}}^{-2}{{\rm{h}}}^{-1}{{\rm{kPa}}}^{-1})={\rm{F}}/{{\rm{P}}}_{0}({\rm{kPa}})$$

where V_0_ is the volume of solvent that passed through the membrane, T_0_ is the time of measurement, A_0_ is the effective membrane area and P_0_ is the applied pressure. The morphology of nanofibrous structure of poly-CD was also evaluated by SEM after the filtration studies. In addition to above mentioned experiments, salt solution (2000 mg/L NaCl) was passed through the system by using the same test parameters. The salt rejection, R (%) of the membrane was evaluated with the following equation:

10 $${\rm{R}}( \% )=(1-{{\rm{C}}}_{{\rm{p}}}/{{\rm{C}}}_{{\rm{f}}})\times 100$$

where C_f_ and C_p_ are the salt concentrations in the feed and permeate solution, respectively. The salt permeability B of the poly-CD membrane was determined according to the following formula:

11 $${\rm{B}}=(1/{\rm{R}}-1)\times {\rm{F}}$$

### Data availability

All data are available from the authors on reasonable request.

## Electronic supplementary material

Supplementary Info

Supplementary Video
