# Supplementary material for: Electrospun crosslinked poly-cyclodextrin nanofibers: Highly efficient molecular filtration thru host-guest inclusion complexation
Source: Sci Rep. 2017 Aug 7;7:7369. doi: 10.1038/s41598-017-07547-4 (PMC5547107; doi:10.1038/s41598-017-07547-4)
Supplement: Supplementary file 1 — Supplementary Info [file 41598_2017_7547_MOESM1_ESM.pdf]

Supporting Information

Electrospun crosslinked poly-cyclodextrin nanofibers: Highly efficient molecular filtration thru host-guest inclusion complexation

Asli Celebioglu[1,2*], Zehra Irem Yildiz[1,2] and Tamer Uyar[1,2*]

Institute of Materials Science & Nanotechnology, UNAM-National Nanotechnology Research Center, Bilkent University, 06800, Ankara, TURKEY

*Corresponding Authors: A.C. (email: celebioglu@unam.bilkent.edu.tr) and T.U. (email: uyar@unam.bilkent.edu.tr)

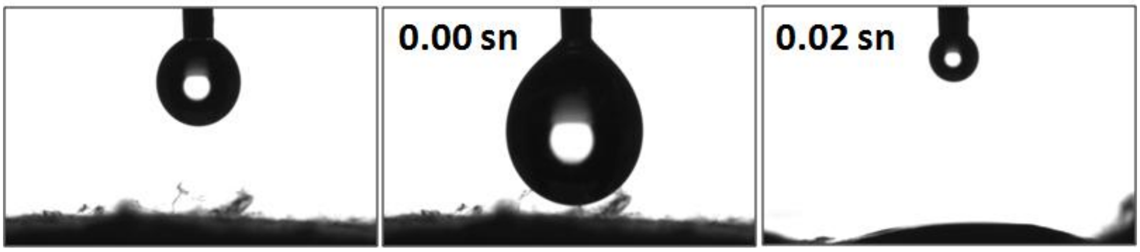

Figure S1. Dynamic contact angle measurement supported the wetting tendency of poly-CD nanofibers and water droplets disappeared just in 0.02 s

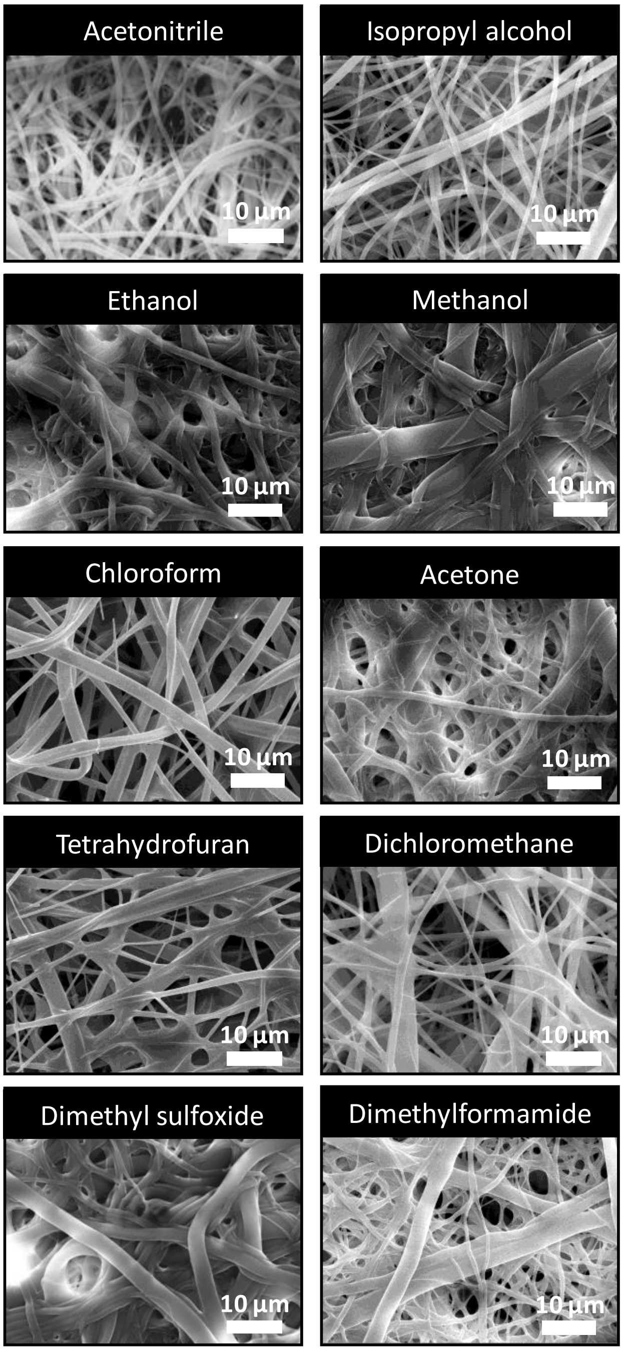

Figure S2. The SEM images of poly-CD nanofibers after soaking them in acetonitrile, isopropanol, ethanol, methanol, chloroform, acetone, tetrahydrofuran, dimethyl sulfoxide (DMSO) and dimethylformamide (DMF) for 24 hours. Poly-CD nanofibers are able to protect their fiber structure even in these strong organic solvents.

Table S1. Fitting parameters of the C 1s XPS spectra of pure CD NF and poly-CD NF/After TT. The peak ratio of O-C-O decrease significantly after TT and the ratio appear for O=C-O due to the positioned cross-linker through fiber structure.

Samples	Bonds	Peak binding energy (eV)	Area ratio (%)
**CD NF**	C-(C-H)	284.70	19.5
	C-O	286.31	61.0
	O-C-O	287.68	19.5
**Poly-CD NF/After TT**	C-(C-H)	284.52	25.3
	C-O	286.02	57.4
	O-C-O	287.36	5.2
	O-C=O	288.36	12.1

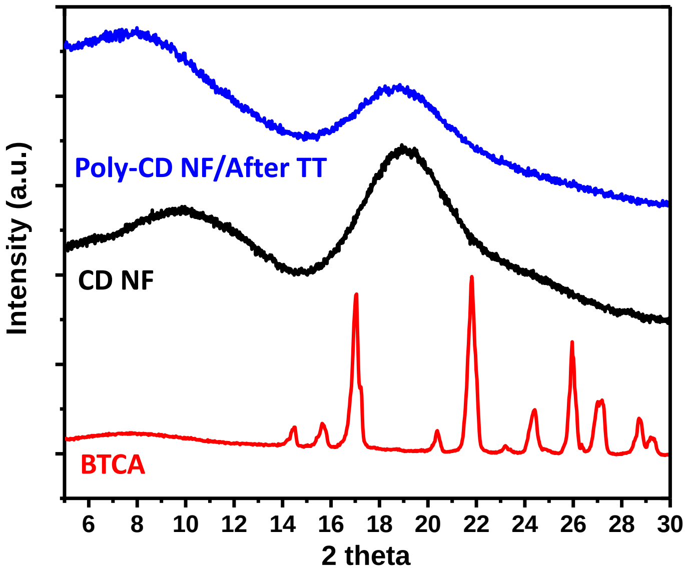

Figure S3. XRD graphs of BTCA, pure CD NF and poly-CD NF/After TT.

Table S2. The tensile test results of electrospun poly-CD nanofibrous web and non-polymeric pure CD nanofibrous web.

Samples	Young Modulus (MPa)	Ultimate Stress (Tensile Strenght) (MPa)	Elongation at Break (%)
poly-CD nanofibrous web	32.60±2.77	0.67±0.06	2.50±0.50
pure CD nanofibrous web*	2.42±1.60	0.16±0.07	3.47±0.55

* The results are obtained from previous study; A. Celebioglu, H. Sener Sen, E. Durgun and T. Uyar, Molecular Entrapment of Volatile Organic Compounds (VOCs) by Electrospun Cyclodextrin Nanofibers, Chemosphere 144 (2016) 736–744.

Table S3. The BET analysis results of poly-CD nanofibrous web

Surface Area (m^2/g)	Total pore volume (cm^3/g)	Average pore width (nm)
6.45	0.16	85.60

Table S4. Kinetics Parameters for the adsorption of MB by poly-CD nanofibrous membrane.

Pollutant	Experimental	Pseudo-first order model			Pseudo-second order model		
	q_{exp} (mg/g)	q_e (mg/g)	k_1 (min^{-1})	R^2	q_e (mg/g)	k_2 ($gmg^{-1}min^{-1}$)	R^2
**MB**	38.472	14.227	0.0044	0.936	39.167	4.5 X10^{-3}	0.9999

note S1: In this study, we have performed batch removal test by using testing solution volume (V) to adsorbent weight ratio; 5mL/5mg. Depending on this, we have obtained experimental q_e (mg/g) value mentioned in Table S4. For an accurate comparison of MB adsorption with the literature, we have also investigated the performance of poly-CD nanofibers by using 40 mg/L concentrated MB solutions and 80mL/14mg ratio as with reports by Zhao et al [12]. After 360 min equilibrium time, we have observed that, MB absorption capacity of poly-CD nanofibers is 125.36 mg/g for the given parameters and it is higher than the compared CD based nanofibers (119.84 mg/g) in the Zhao study, even we did not arrange optimum pH. Therefore, we can conveniently claim that, poly-CD nanofibers remove MB more efficiently compared to *"β-Cyclodextrin-Based Electrospun Nanofiber"* which was declared as the best adsorbent among other available cyclodextrin based materials reported the literature [12].

Table S5. Langmuir and Freundlich isotherm model parameters for MB adsorptions by poly-CD nanofibrous membrane and activated carbon (AC).

MB (concentration range)	Absorbent	Langmuir isotherm			Freundlich isotherm		
		q_{max} (mg/g)	b (L/mg)	Ry^2	K_F	1/n	Ry^2
MB (0-600 mg/L)	Poly-CD membrane	97.43	0.5618	0.9983	34.02	0.2047	0.8532
MB (200-600 mg/L)	Poly-CD membrane (pH=7)	96.79	-1.007	0.9975	78.28	0.0378	0.2681
MB (200-600 mg/L)	AC	14.73	1.4357	0.7462	860.476	-0.6631	0.4408
MB (200-600 mg/L)	Poly-CD membrane (pH=9)	124.102	0.3053	0.9996	103.760	0.027990	0.8907

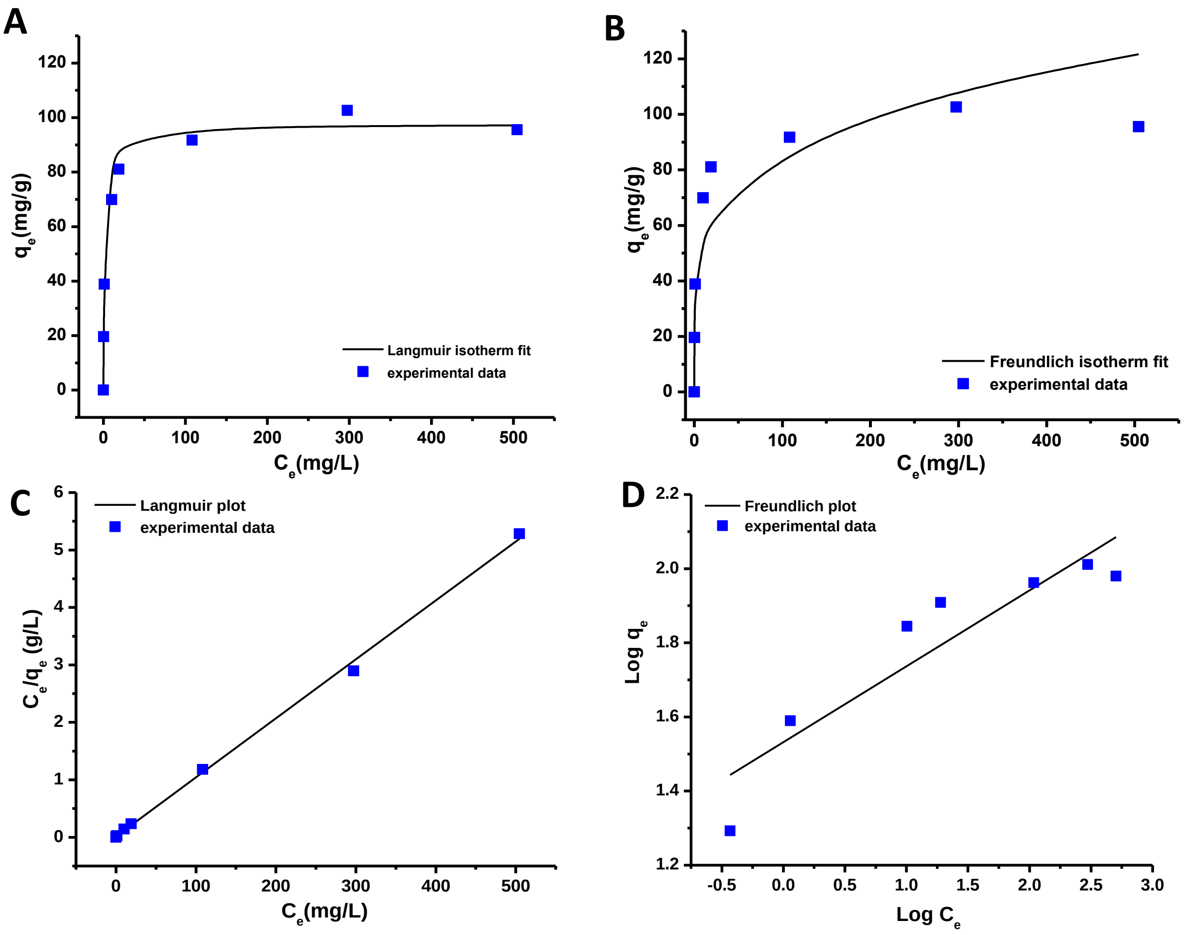

Figure S4. Adsorption isotherms **(a-b)** and the corresponding Langmuir plot **(c)** and Freundlich plot **(d)** for MB adsorption by nanofibrous membrane.

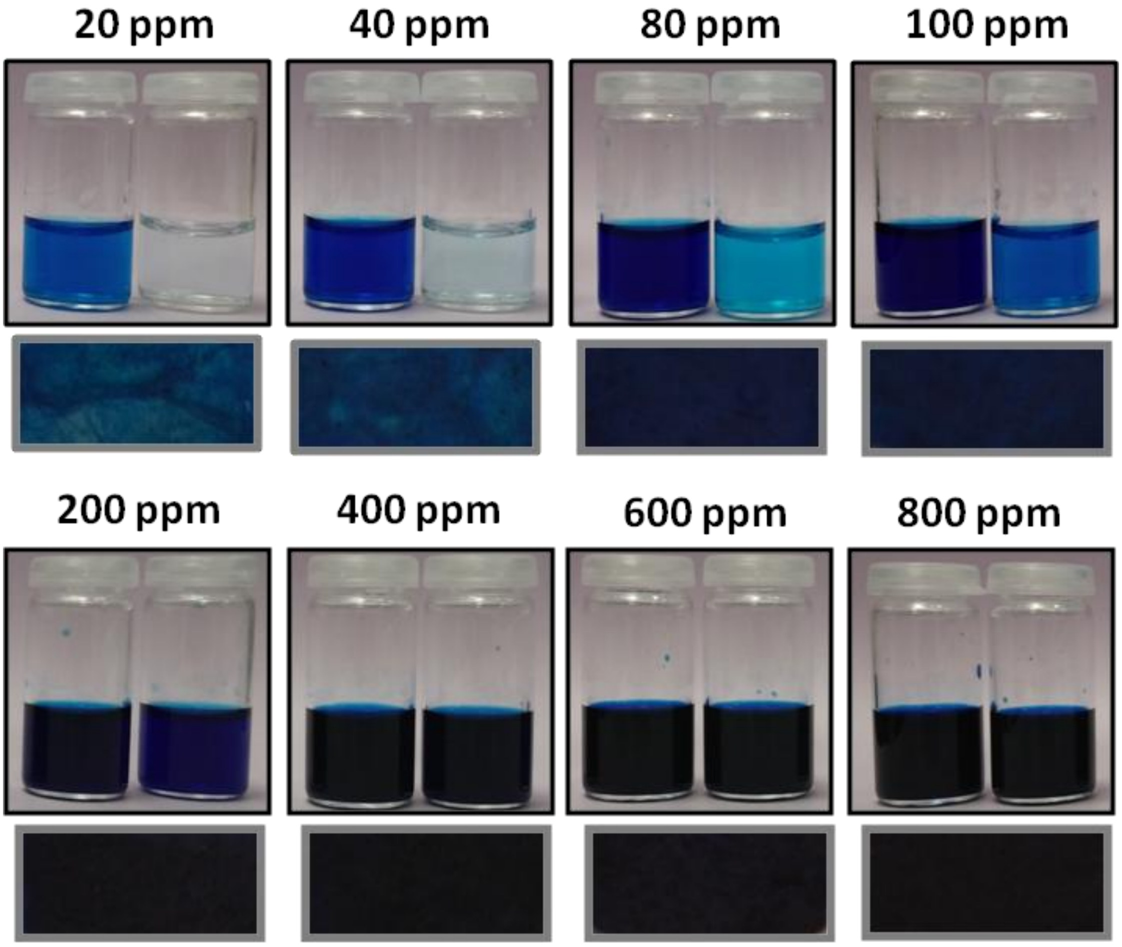

Figure S5. Visual representation of the color changes of both MB solutions and poly-CD nanofibrous membrane depending on the increasing MB concentration during the removal tests. As it is observed the color of the poly-CD nanofibrous membrane is getting darker for the higher MB concentration by the adsorption of much more MB molecules.

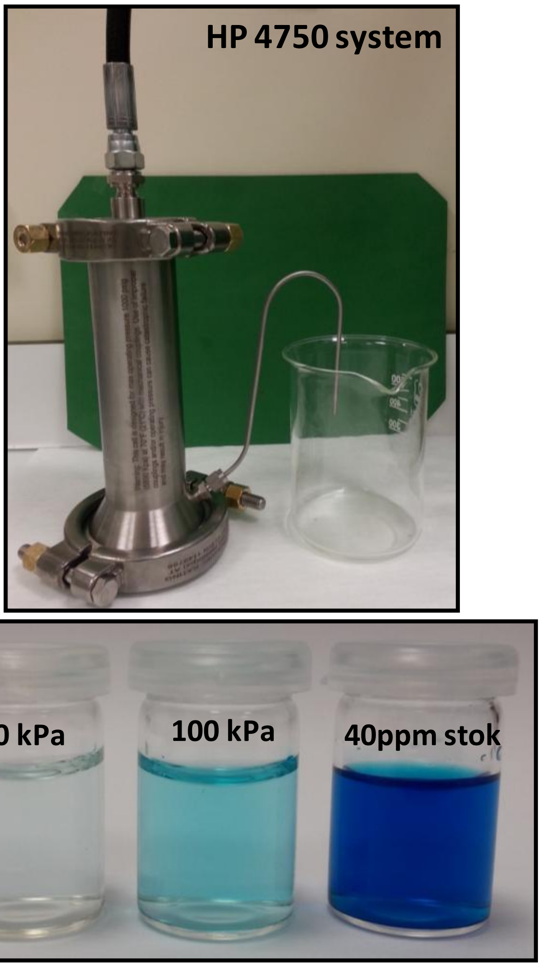

Figure S6. The photograph of dead-end system (Sterlitech HP4750) used during the filtration experiments. For this, poly-CD nanofibrous membrane were located as a thin layer into membrane cell. 50 mL pollutant solution was passed rapidly through the poly-CD nanofibrous membranes (active filtration area; 14.6 cm^2) by applying N_2 pressure. Finally, filtered solutions are collected into a clear beaker for further analysis. To investigate the pressure effect on filtration performance, two pressure values; 100kPa and 10kPa were applied to the 40 mg/L concentrated MB solutions. As it is observed, better adsorption is provided by operating 10kPa, since the flux values increase significantly in case of 100kPa (for 10kPa; 3842 $Lm^{-2}h^{-1}$ and 100kPa; 13698 $Lm^{-2}h^{-1}$), this decreases the penetration time of the solution and depending on this, the removal efficiency of MB decreases.

Table S6. The conditions of the dead-end filtration systems (HP4750). The removal efficiency (%) and the SEM images of poly-CD nanofibrous membrane as results of filtration experiments.

**Pollutant (concentration)**	**Permeability ($Lm^{-2}h^{-1}kPa^{-1}$)**	**Flux ($Lm^{-2}h^{-1}$)**	**Removal efficiency (%)**	**SEM images of poly-CD nanowebs after filtration test**
**MB (40 mg/L)**	291±54	3842±413	98.6±0.4	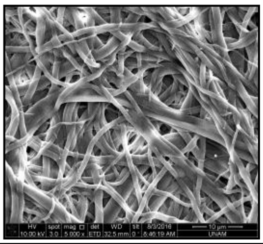
**MB (80 mg/L)**	237±40	3561±450	95.6±0.9	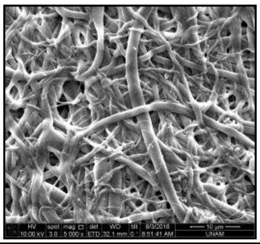

movie S1. The video of the removal of MB solution by dead-end filtration system under high flux.

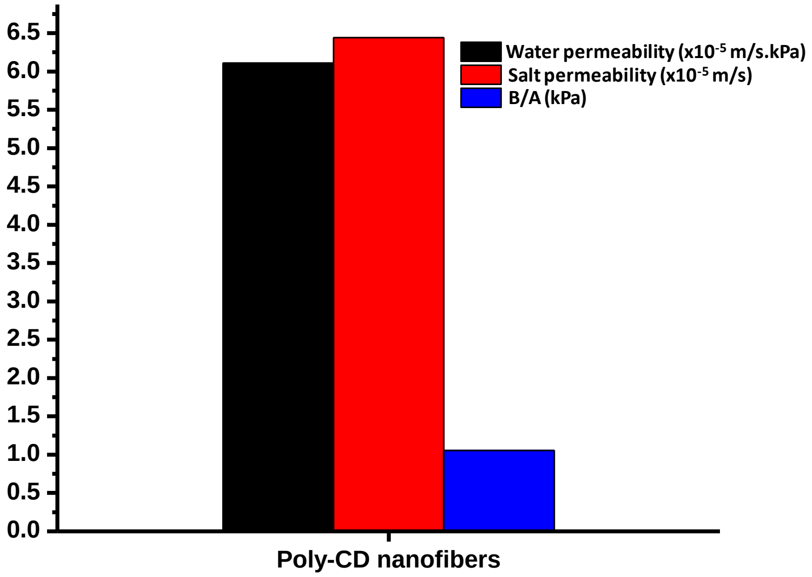

Figure S7. Water permeability and salt permeability graphs of poly-CD nanofibrous membrane and B/A value which is calculated from the ratio of these two parameters (salt permeability/water permeability). The low B/A ratio indicates the selectivity and applicability of membranes without an accumulation of salt. We have obtained B/A ratio as 1.05 kPa and this is quite less than the value reported in the literature [32, 33].
